# Poly(arylene ether nitrile) Composites with Surface-Hydroxylated Calcium Copper Titanate Particles for High-Temperature-Resistant Dielectric Applications

**DOI:** 10.3390/polym11050766

**Published:** 2019-05-01

**Authors:** Junyi Yang, Zili Tang, Hang Yin, Yan Liu, Ling Wang, Hailong Tang, Youbing Li

**Affiliations:** 1 College of Materials Science and Engineering, Chongqing University of Technology, Chongqing 400054, China; yangjunyiaiai@163.com (J.Y.); T13308240641@163.com (Z.T.); 15838190312@163.com (H.Y.); liuyaner1116@163.com (Y.L.); 13996584721@163.com (L.W.); lyb123@cqut.edu.cn (Y.L.); 2 Chongqing Key Laboratory of Mold Technology, Chongqing University of Technology, Chongqing 400054, China

**Keywords:** poly(arylene ether nitrile), composites, calcium copper titanate, dielectric properties, high-temperature-resistant

## Abstract

In order to develop high-performance dielectric materials, poly(arylene ether nitrile)-based composites were fabricated by employing surface-hydroxylated calcium copper titanate (CCTO) particles. The results indicated that the surface hydroxylation of CCTO effectively improved the interfacial compatibility between inorganic fillers and the polymer matrix. The composites exhibit not only high glass transition temperatures and an excellent thermal stability, but also excellent flexibility and good mechanical properties, with a tensile strength over 60 MPa. Furthermore, the composites possess enhanced permittivity, relatively low loss tangent, good permittivity-frequency stability and dielectric-temperature stability under 160 °C. Therefore, it furnishes an effective path to acquire high-temperature-resistant dielectric materials for various engineering applications.

## 1. Introduction

In recent decades, polymer-based composites with high dielectric permittivity and low loss tangents have received extensive attention in aerospace, microelectronic devices and electronic components such as film capacitors, embedded capacitors [1,2,3,4,5]. Although polymers have good mechanical properties and processability, their dielectric properties are not ideal. As far as we know, most ceramic fillers have good dielectric properties, such as high dielectric permittivity and low loss tangent [6,7]. Therefore, polymer-based composites have been developed to combine the advantages of polymers and ceramic fillers, which possess high dielectric permittivity and low loss tangent as well as good processability. Generally, fillers for dielectric composites are classified into conductive fillers (e.g., graphene [8,9,10,11,12,13], carbon nanotubes [14,15,16,17,18,19]) and dielectric fillers (e.g., barium titanate [20,21,22], strontium titanate [23]). Although polymer-based composites with conductive fillers have a large increase in dielectric permittivity, their loss tangents are also greatly increased. Moreover, when the content of the conductive fillers increases to a certain value (i.e., the percolation threshold), the loss tangent will increase exponentially, which is a fatal disadvantage for their practical applications.

As a typical dielectric filler, calcium copper titanate (CCTO) has a high dielectric constant, low loss tangent and low-temperature dependency of dielectric permittivity [24,25]. However, there are two problems to be solved in order to fabricate polymer-based CCTO composites with excellent performance. Firstly, the poor compatibility between the inorganic fillers and the polymer matrix [26,27] needs to be improved. Secondly, the spontaneous aggregation of the fillers is another problem that will affect the performance of the composite. Dang et al. [28] achieved high dielectric permittivity and good thermal stability in functional hybrid films by employing giant dielectric permittivity calcium copper titanate as a functional inorganic filler and thermosetting polyimide as a polymer matrix. Tang et al. [29] prepared poly(arylene ether nitrile) nanocomposites by employing core-shell structured BaTiO_3_@polymer nanoparticles as hybrid nanofillers, which exhibited enhanced dielectric properties, good permittivity-frequency stability, excellent thermal stability, high mechanical strength and good flexibility.

As a high-performance engineering plastic, poly(arylene ether nitrile)s (PAENs) have excellent mechanical properties, thermal properties, chemical resistance and easier processability [30,31,32,33,34,35]. Therefore, PAENs can be attractive candidates as polymer matrices in advanced functional materials. In this work, we performed the surface hydroxylation of CCTO to obtain hydroxylated CCTO (h-CCTO) particles, and then developed PAEN-based composites by employing h-CCTO particles as fillers. Furthermore, the mechanical properties, thermal properties and microscopic morphology of the composites were characterized in detail, and their dielectric properties were intensively investigated.

## 2. Materials and Methods

### 2.1. Materials

Poly(arylene ether nitrile) was synthesized by the nucleophilic aromatic substitution polymerization of 2, 6-dichlorobenzonitrile and bisphenol A in *N*-methyl-2-pyrrolidone (NMP) medium with anhydrous potassium carbonate as a catalyst, according to the method in our previous literature [36]. The corresponding schematic procedure is shown in Figure S1. Calcium copper titanate particles (the size distribution is about 150–300 nm) were supplied by Shanghai Dian Yang Industry Co., Ltd., Shanghai, China. *N*-methyl-2-pyrrolidone (NMP) was purchased from Shanghai Titan Scientific Co., Ltd., Shanghai, China. Hydrogen peroxide (H_2_O_2_) was provided by Chengdu Jinshan Chemical Reagent Co., Ltd., Chengdu, China.

### 2.2. Surface Hydroxylation of CCTO Particles

Calcium copper titanate was chosen as the filler due to its giant dielectric permittivity and low dielectric loss. To prepare the surface hydroxylated CCTO particles, the pristine CCTO were ultrasonically dispersed in an aqueous solution of H_2_O_2_ (30%, 150 mL) for 0.5 h and refluxed at 105 °C for 4 h while being stirred vigorously, and then it was ultrasonically dispersed for 15 min. After centrifugation, the upper layer of the clarified liquid is sucked out with a dropper, and the lower mixture was washed with deionized water for five times. Finally, h-CCTO particles were obtained after drying in a vacuum oven at 60 °C for 24 h.

### 2.3. Preparation of PAEN/h-CCTO Composite Films

PAEN/h-CCTO composite films were fabricated by the continuous ultrasonic dispersion technique and solution casting method. Firstly, a certain amount of h-CCTO particles were dispersed in NMP under violent ultrasonic treatment accompanying with vigorous stirring. Meanwhile, the PAEN was dissolved in NMP by heating and stirring. Afterward, the h-CCTO particles suspension was added slowly into the PAEN solution in an ultrasonic water bath at 60 °C with mechanical stirring for 1 h to ensure that the h-CCTO particles were uniformly dispersed in the PAEN solution. Simultaneously, a clean glass plate was put into the oven and kept horizontal. Finally, the homogeneous suspension was cast slowly on the glass plate and then dried in an oven at 60 °C, 80 °C, 120 °C and 160 °C (each for 2 h) to thoroughly evaporate the solvent. After naturally cooling to room temperature, PAEN/h-CCTO composite films with different mass fractions of h-CCTO (0 wt%, 15 wt%, 30 wt%, 45 wt% and 60 wt%) were obtained.

### 2.4. Characterizations

Fourier transform infrared (FTIR) spectrum was recorded on a Nicolet iS10 FTIR spectrometer (Thermo Scientific, Waltham, MA, USA) by using the KBr pellet method. Scanning electron microscope (SEM) was performed on a Zeiss SIGMA HDTM field emission gun scanning electron microscope at 20 kV. SEM samples were fractured in liquid nitrogen and then sputtered with gold on the fracture surface. Thermogravimetric analysis (TGA) was operated on a TGA-Q50 (TA Instruments, New Castle, DE, USA) at a heating rate of 20 °C/min from room temperature to 800 °C. Differential scanning calorimetry (DSC) measurements were measured using a DSC-Q20 (TA Instruments) system at a heating rate of 10 °C /min from 30 °C to 200 °C under flowing nitrogen. A mechanical test was carried out on a SANS CMT6104 Series Desktop Electromechanical Universal Testing Machine. Dielectric properties of PAEN/h-CCTO composites were measured by the Partulab HDMS-1000 high-temperature dielectric measurement system (Wuhan Partulab Technology, Wuhan, China) combined with an Agilent 4294A Precision Impedance Analyzer (Agilent Technologies, Santa Clara, CA, USA). For dielectric measurements, the composite films (thickness: 0.03–0.05 mm) were cut into small square samples (12 mm × 12 mm) and then coated with colloidal silver electrodes on both sides.

## 3. Results and Discussion

### 3.1. Surface Hydroxylation of CCTO Particles

**Figure 1:**
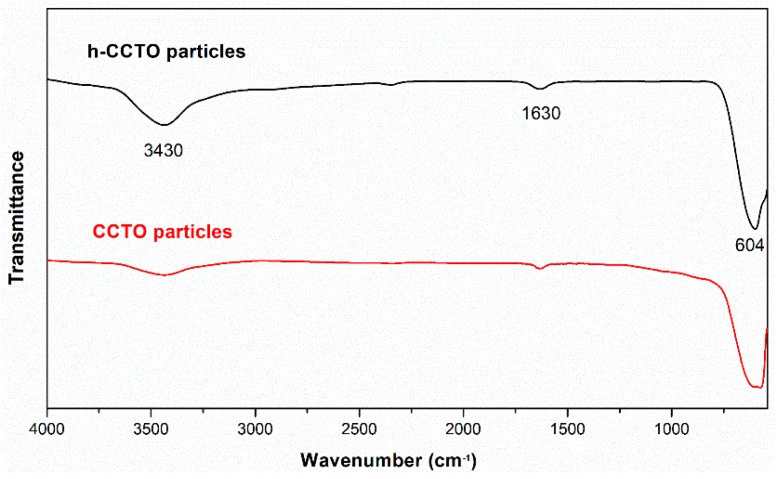
The Fourier transform infrared (FTIR) spectra of calcium copper titanate (CCTO) and hydroxylated CCTO (h-CCTO) particles.

**Figure 2:**
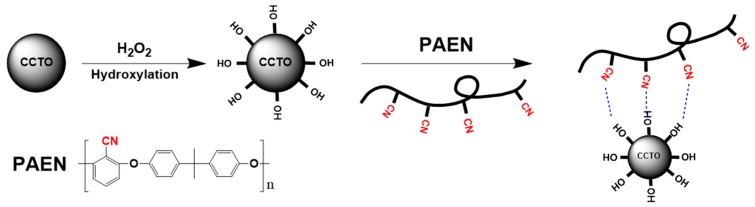
The schematic diagram of the hydroxylation of CCTO particles and interactions between h-CCTO particles and poly(arylene ether nitrile)s (PAEN) matrix.

As reported in the literature [37], H_2_O_2_ treatment is a simple and efficient method to derive massive hydroxy groups on the surface of inorganic particles. The effect of H_2_O_2_ treatment on the surface chemistry of CCTO particles was studied by FTIR. As shown in Figure 1, the band at 604 cm^−1^ corresponds to the Ti–O vibration of CCTO, and the weak peak at 1630 cm^−1^ may be attributed to the bending mode of H–O–H, resulting from the physically adsorbed water. In addition, a broad and strong band at 3430 cm^−1^ was observed in the FTIR spectrum of h-CCTO particles, which was assigned to the stretching mode of O–H. As a comparison, a relatively weak band at 3430 cm^−1^ was obtained in the FTIR spectrum of CCTO particles, which might be due to a small amount of physically adsorbed water. These results indicate that the CCTO particles after surface hydroxylation contain a large number of hydroxyl groups on the particles’ surface. This can enhance the compatibility between the organic-inorganic interface, due to the fact that it can produce strong dipole-dipole interactions between the polar hydroxyl groups and nitrile groups, as schemed in Figure 2. Furthermore, it is also beneficial to improve the dispersibility of the particles in the polymer matrix, which can be verified by the SEM images and dispersion photograph of CCTO and h-CCTO particles, as shown in Figures S2 and S3, respectively. It was found that less and smaller particle aggregates were formed after surface hydroxylation of CCTO particles.

### 3.2. Morphologies of PAEN/h-CCTO Composites

**Figure 3:**
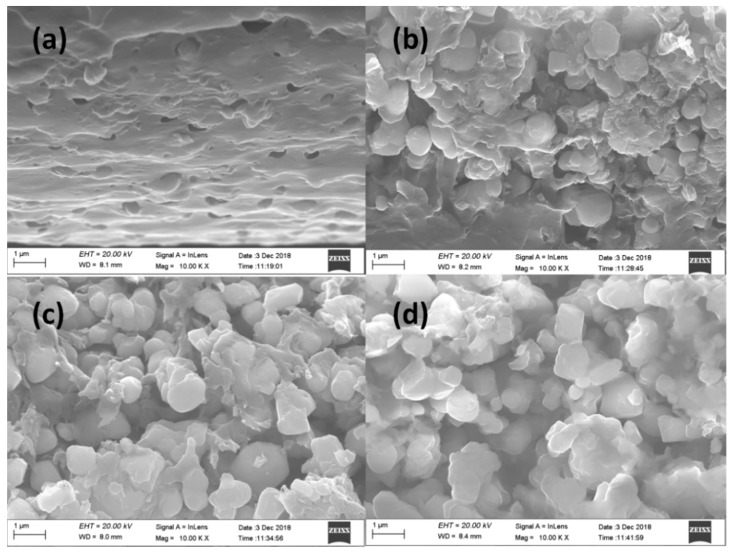
The cross-sectional scanning electron microscope (SEM) images of PAEN/h-CCTO composite films: (**a**) 15 wt%, (**b**) 30 wt%, (**c**) 45 wt%, (**d**) 60 wt%.

The cross-sectional morphologies of the PAEN/h-CCTO composite films were investigated by SEM. As shown in Figure 3, it is obvious that the dispersion density of particles increases with the increasing mass fraction of h-CCTO. The particles are relatively uniformly dispersed in the PAEN matrix for the composite with 15 wt% h-CCTO content. However, some partial agglomerations of particles were found for the composites with high h-CCTO loadings (i.e., 30 wt%, 45 wt% and 60 wt%). Furthermore, the phase interfaces between the particles and the PAEN matrix are almost indistinguishable, which indicates good compatibility between h-CCTO particles and the PAEN matrix. This may be attributed to the enhanced intermolecular interactions between h-CCTO particles and PAEN matrix through the strong dipole-dipole interactions of hydroxyl groups and nitrile groups. As a contrast, the cross-sectional SEM image of PAEN composite with 15 wt% non-hydroxylated CCTO particles is shown in Figure S4 (Supplementary Information). It was found that a lot of holes arising from particle extractions are distinctly visible, and the phase interface between CCTO particles and the PAEN matrix is very clear, which indicates that the compatibility between non-hydroxylated CCTO particles and the PAEN matrix is very poor. These results further verify that the surface hydroxylation can effectively improve the interfacial compatibility between CCTO particles and the PAEN matrix.

### 3.3. Thermal Properties of PAEN/h-CCTO Composites

**Figure 4:**
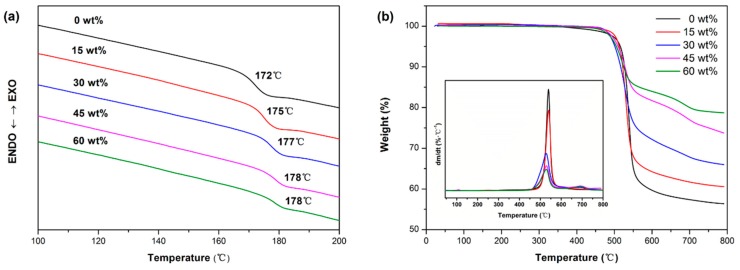
(**a**) The differential scanning calorimetry (DSC) and (**b**) thermogravimetric analysis (TGA) and derivative thermogravimetry (DTG) curves of PAEN/h-CCTO composites.

As a high-performance engineering plastic, PAEN possesses excellent thermal properties. As shown in Figure 4, the thermally induced phase transition and thermal decomposition behaviors of PAEN/h-CCTO composites were examined by DSC and TGA under a nitrogen atmosphere, respectively. Their glass transition temperatures (*T_g_*s), decomposition temperatures at 5% weight loss (*T_5%_*s), temperatures of the maximum decomposition rate (*T_max_*s) and char yields (*CY*s) are summarized in Table 1. As shown in Figure 4a, all the composites have a pretty high *T_g_*, and their *T_g_*s were found to gradually increase from 172 °C to 178 °C when the h-CCTO loadings increased from 0 to 60 wt%. This may be due to the fact that the rigid particles hinder the movement of the molecular segments.

As can be seen in Figure 4b and Table 1, It is obvious that all the PAEN/h-CCTO composites have a high *T_5%_* (over 500 °C), with a slight variation around 510 °C, and their *T_max_*s are around 530 °C. Furthermore, the *CY*s at 800 °C were found to gradually increase with the progressive increase of h-CCTO contents, from 56.4% for pure PAEN to 78.7% for the composite with 60 wt% h-CCTO content. This is consistent with the amount of h-CCTO loadings in the composites. The above results indicate that the PAEN/h-CCTO composites have high *T_g_* and excellent thermal stability, which is mainly attributed to the excellent thermal properties of the PAEN matrix, and thus the composites might be a good application prospect in high-temperature-resistant electronic components.

### 3.4. Dielectric Properties of PAEN/h-CCTO Composites

**Figure 5:**
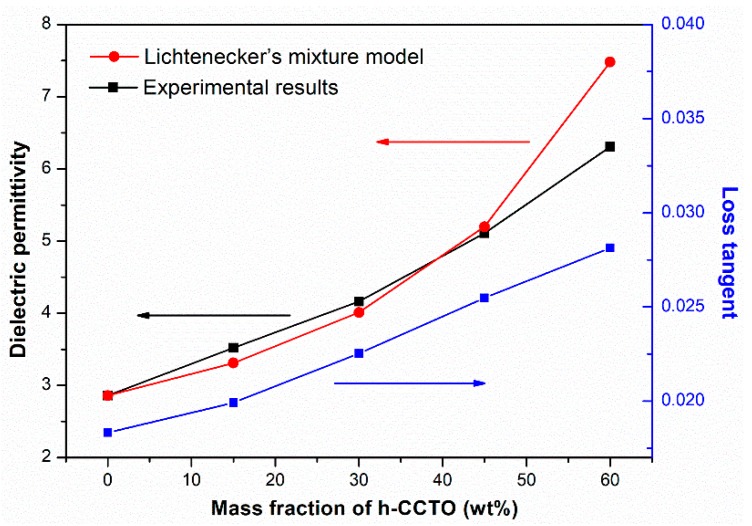
The experimental dielectric properties of PAEN/h-CCTO composites as a function of the h-CCTO content at 1 kHz and theoretical dielectric permittivity based on Lichtenecker’s logarithmic mixture model.

As shown in Figure 5, the dielectric permittivity and loss tangent of PAEN/h-CCTO composites were measured at 1 kHz to discern the effect of the h-CCTO content on their dielectric properties. As we can see, the dielectric permittivity gradually increases with the increase of the h-CCTO content. When the mass fraction of h-CCTO increases from 0 wt% to 60 wt%, the dielectric permittivity increases from 2.86 to 6.31. Compared with pure PAEN, the dielectric permittivity of the composite with 60 wt% h-CCTO loading increased by about 121%. To further illustrate the effect of fillers on the dielectric properties of composites, theoretical calculations were conducted based on Lichtenecker’s logarithmic mixture model [38], as summarized in the Supplementary Information (Section S2 and Table S1). The comparison of experimental and theoretical dielectric permittivity is also shown in Figure 5. As we can see, when the mass fraction of h-CCTO particles increased from 0 to 45 wt%, the experimental dielectric permittivities are in good agreement with the theoretical values calculated by Lichtenecker’s logarithmic mixture model. However, for the composite with 60 wt% h-CCTO content, the experimental dielectric permittivity is lower than the theoretical value. This may be caused by a decrease in interfacial polarizations due to the more serious agglomeration in the composite with high particle content. Loss tangent is also an important parameter for dielectric materials. Generally, composites with a higher loss tangent tend to dissipate more energy in the form of heat, which is detrimental to the application in electronic components. Although the loss tangent of the composites increases with the increase of the h-CCTO content, from 0.018 for pure PAEN to 0.028 for the composite with 60 wt% h-CCTO loading, it is still in a relatively low range for practical applications. The low loss tangent is mainly attributed to the good compatibility between the particles and the polymer matrix, which leads to a reduction of the loss tangent derived from interfacial polarization.

**Figure 6:**
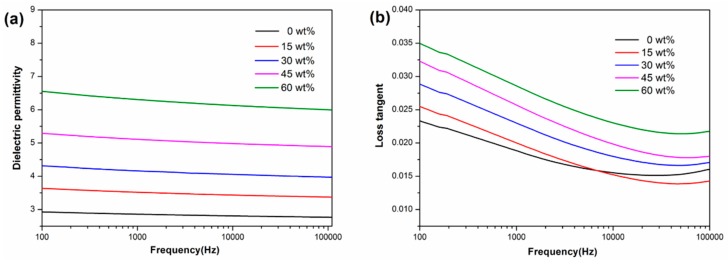
(**a**) The dielectric permittivity and (**b**) loss tangent of PAEN/h-CCTO composites as a function of frequency from 100 Hz to 100 kHz.

Moreover, the dielectric properties were measured as functions of frequency and temperature to discern their frequency-dependence and temperature-dependence, respectively. As shown in Figure 6, the dielectric permittivity of the composites has a slight decrease with the increasing frequency, with a fluctuation range within ± 6% over the frequency from 100 Hz to 100 kHz. The loss tangent of the composites demonstrates a decrease with the increase of frequency from 100 Hz to 20 kHz, and then presents a slight increase at frequencies exceeding 20 kHz. Overall, the PAEN/h-CCTO composites possess a good permittivity-frequency stability and a relatively low loss tangent.

**Figure 7:**
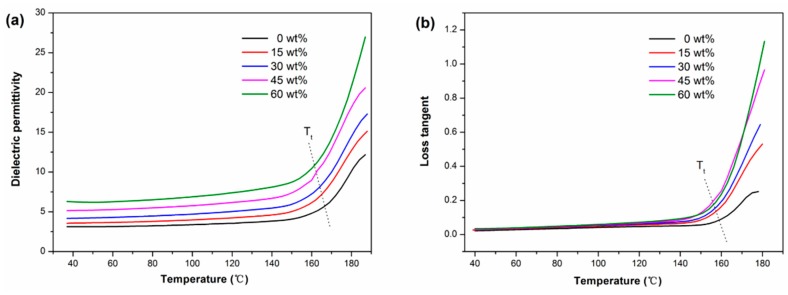
(**a**) The dielectric permittivity and (**b**) loss tangent of PAEN/h-CCTO composites as a function of temperature from 40 °C to 180 °C.

Figure 7 depicts the dielectric permittivity and loss tangent of PAEN/h-CCTO composites as a function of temperature from 40 °C to 180 °C at 1 kHz. It was found that the dielectric permittivity and loss tangent were relatively stable when the temperature is below the turning point temperature (*T_t_*). When the temperature reaches and exceeds the turning point temperature, both the dielectric permittivity and loss tangent display a rapid increase. It is important to note that the turning point temperatures (160~170 °C) are close to their glass transition temperatures (around 175 °C), which suggests that the *T_g_* of polymer matrix plays a dominant role in determining the dielectric-temperature stability of polymer-based composites. This phenomenon might be attributed to polymer molecular motions. When the temperature is below *T_g_*, there are mainly local motions of the side groups on the macromolecular chains, and these motions are relatively weak. However, when the temperature reaches and exceeds the *T_g_*, the motion intensity of the side groups is greatly increased, accompanied by more intense segmental movements of macromolecular chains. This will lead to the rapid increase of molecular polarizations (contribution to dielectric permittivity) and conduction loss (contribution to a loss tangent), which can be further verified by the relationship of electrical conductivity of the composite versus temperature (Figure S5 in Supplementary Information). The above results indicate that the PAEN/h-CCTO composites possess good dielectric-temperature stability under 160 °C, which is greatly significant for their potential applications as high-temperature-resistant electronic components.

### 3.5. Mechanical Properties and Flexibility of PAEN/h-CCTO Composites

**Figure 8:**
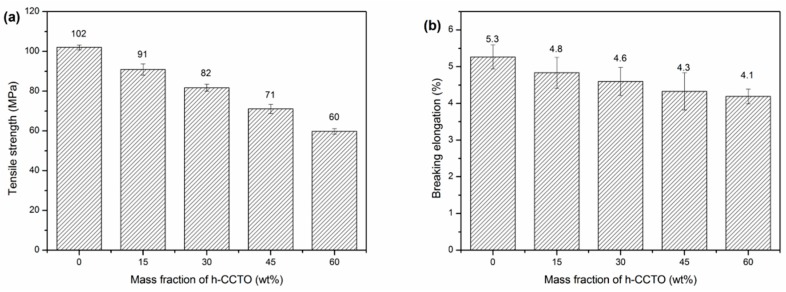
(**a**) The tensile strength and (**b**) breaking elongation of PAEN/h-CCTO composites.

**Figure 9:**
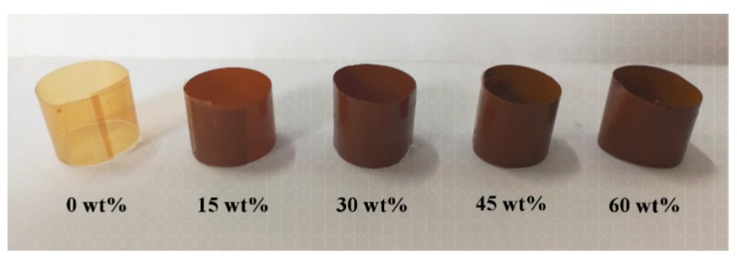
The digital photo of PAEN/h-CCTO composite films curled into multilayer cylinders.

As shown in Figure 8, the tensile strength and breaking elongation of the composites were tested to study the effect of h-CCTO particle loadings on the mechanical properties of the composites. It was found that the tensile strength gradually decreased with the increase of h-CCTO loading, from 102 MPa for the pure PAEN to 60 MPa for the composite with 60 wt% h-CCTO loading. This is due to the partial self-aggregation phenomenon arising from the increase of the particle loading for the composites with a large number of inorganic particles, which is consistent with the SEM observations (Figure 3b–d). Nonetheless, the tensile strength of the PAEN/h-CCTO composites can still be comparable to other ordinary engineering plastics, such as polyamides, polyformaldehyde. As shown in Figure 8b, the breaking elongation also gradually decreased with the increase of h-CCTO loading, ranging from 4.1% to 5.3%. It is worth noting that the composite films can be easily bent and curled into multi-layer cylinders, even for the composite film with 60 wt% h-CCTO content (Figure 9), which is beneficial for their potential application in film capacitors. The above results demonstrate that the PAEN/h-CCTO composites possess a relatively high tensile strength and excellent flexibility, and can fully satisfy the requirements for the practical applications in electronic components. 

## 4. Conclusions

In summary, a series of PAEN/h-CCTO composites were fabricated through continuous ultrasonic dispersion technique and solution casting method by employing surface-hydroxylated CCTO particles, and the surface hydroxylation of CCTO effectively improved the interfacial compatibility between inorganic fillers and the polymer matrix. The results indicated that the composites have high *T_g_*s (over 170 °C) and excellent thermal stability, with *T_5%_*s exceeding 500 °C. Furthermore, the composites exhibited improved dielectric properties, such as enhanced permittivity, relatively low loss tangent and good permittivity-frequency stability, as well as good dielectric-temperature stability under 160 °C. In addition, the composites also exhibited good mechanical properties and excellent flexibility, with a tensile strength over 60 MPa. In view of their excellent overall performances, the composites will have potential applications in the field of high-temperature-resistant electronic components, such as film capacitors, embedded capacitors, etc.

## Figures and Tables

**Table 1:** The data of thermal properties of poly(arylene ether nitrile)s (PAEN)/hydroxylated calcium copper titanate (h-CCTO) composites.

Mass Fraction of h-CCTO	0 wt%	15 wt%	30 wt%	45 wt%	60 wt%
*T_g_* (°C)	172	175	177	178	178
*T_5%_* (°C)	516	511	501	510	505
*T_max_* (°C)	539	531	529	529	522
*CY* (%)	56.4	60.2	66.0	73.7	78.7

## Supplementary Materials

The following are available online at https://www.mdpi.com/2073-4360/11/5/766/s1, Figure S1. Schematic synthesis procedure of poly(arylene ether nitrile). Figure S2. Field emission scanning electron micrograph (FE-SEM) images of (a) CCTO and (b) h-CCTO particles. Figure S3. Digital photo of dispersions of pure CCTO and h-CCTO particles in ethanol after standing for four hours. Figure S4. Cross-sectional SEM image of PAEN composites with 15 wt% non-hydroxylated CCTO particles. Figure S5. Electrical conductivity of PAEN/h-CCTO composite with 30 wt% h-CCTO content as a function of temperature. Figure S6. TGA and DTG curves of h-CCTO particles. Table S1. Experimental and theoretical dielectric permittivity of PAEN/h-CCTO composites.
