# Supplementary material for: Poly(arylene ether nitrile) Composites with Surface-Hydroxylated Calcium Copper Titanate Particles for High-Temperature-Resistant Dielectric Applications
Source: Polymers (Basel). 2019 May 1;11(5):766. doi: 10.3390/polym11050766 (PMC6571784; doi:10.3390/polym11050766)
Supplement: Supplementary file 1 [file polymers-11-00766-s001.pdf]

polymers

MDPI

Supplementary Information: Poly(arylene ether nitrile) Composites with Surface-Hydroxylated Calcium Copper Titanate Particles for High-Temperature-Resistant Dielectric Applications

Junyi Yang, Zili Tang, Hang Yin, Yan Liu, Ling Wang, Hailong Tang and Youbing Li

S1. Synthesis of Poly(arylene ether nitrile) (PAEN)

In a typical synthesis procedure, 2,6-Dichlorobenzonitrile (22.0 g, 128 mmol), bisphenol A (29.2 g, 128 mmol), anhydrous K_2CO_3 (35.4 g, 256 mmol), NMP (85 mL), and toluene (35 mL) were added into a 250 mL three-neck round bottom flask equipped with a Dean-Stark trap, condenser, mechanical stirrer, and thermometer. The system was heated to 140–160 °C to remove water from the reaction by azeotropic distillation with toluene for 3 h. Toluene was then removed by distillation and the temperature was gradually raised to 190–200 °C. The system was kept stirring for about 5 h until its viscosity did not increase any more. Afterwards, the reaction mixture was poured into deionized water to precipitate the polymer. The precipitate was crushed and then poured into 1 L of diluted HCl solution in order to remove the excess K_2CO_3. Finally, the collected polymer was washed five times with boiling deionized water and dried in a vacuum oven at 130 °C for 12 h to produce a white solid.

S2. Theoretical Calculation by Lichtenecker's Logarithmic Mixture Model

The relationship between the dielectric properties of a two-phase mixture and those of each phase in the mixture can be expressed by Lichtenecker's logarithmic mixture model [1].

$$\log \varepsilon = f_1 \log \varepsilon_1 + f_2 \log \varepsilon_2 \qquad (1)$$

where ε is the complex permittivity of the composites, ε_1 and f_1 are the dielectric permittivity and volume fraction of the polymer matrix, and ε_2 and f_2 are the dielectric permittivity and volume fraction of the inorganic filler, respectively.

Dielectric permittivity of PAEN: ε_1 = 2.86 (1 kHz) Density of PAEN: ϱ(PAEN) = 1.18

Dielectric permittivity of h-CCTO: ε_2 =114 (1 kHz) Density of h-CCTO: ϱ(h-CCTO) = 5

For example: PAEN/h-CCTO composites with 15 wt% h-CCTO particles

Assumed total mass: m = 100

m(PAEN) = 85 m(h-CCTO) = 15

V(PAEN) = m(PAEN) / ϱ(PAEN) = 72

V(h-CCTO) = m(h-CCTO) / ϱ(h-CCTO) = 3

$$f_1 = \frac{V(PAEN)}{V(PAEN) + V(\text{h-CCTO})} = 0.96$$

$f_2 = 1 - f_1 = 0.04$

Calculated by Equation (1): ε = 3.31

Table S1. Experimental and theoretical dielectric permittivity of PAEN/h-CCTO composites.

Mass fraction of h-CCTO	0 wt%	15 wt%	30 wt%	45 wt%	60 wt%
Experimental values	2.86	3.52	4.16	5.11	6.31
Theoretical values	2.86	3.31	4.01	5.20	7.48

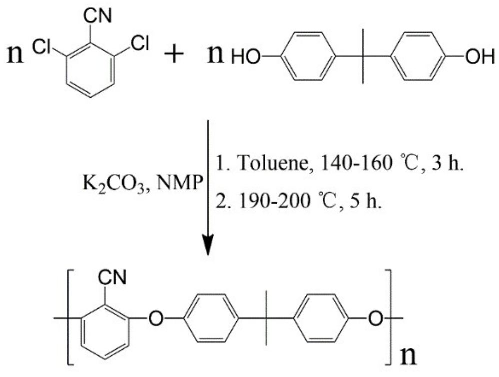

Figure S1. Schematic synthesis procedure of poly(arylene ether nitrile).

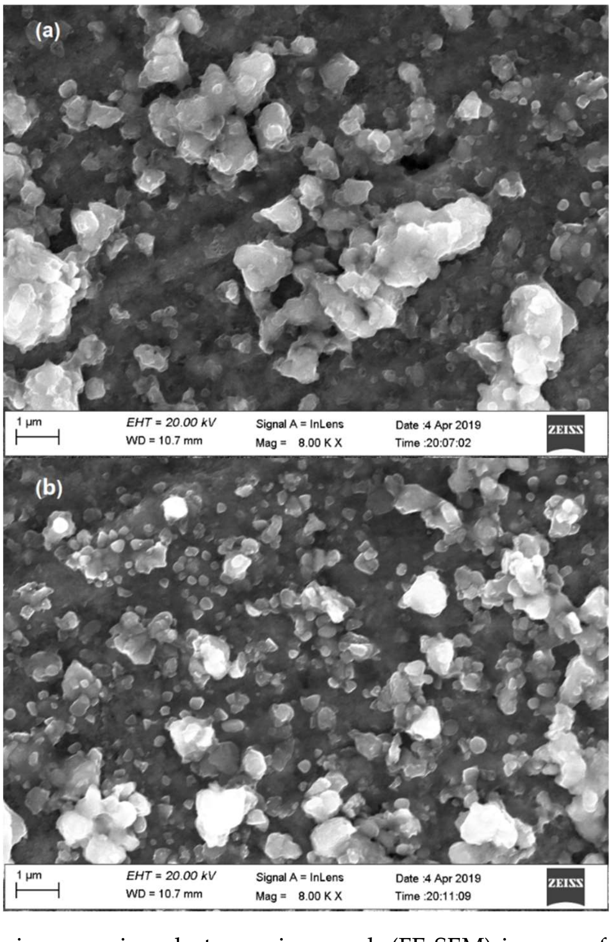

Figure S2. Field emission scanning electron micrograph (FE-SEM) images of (**a**) CCTO and (**b**) h-CCTO particles.

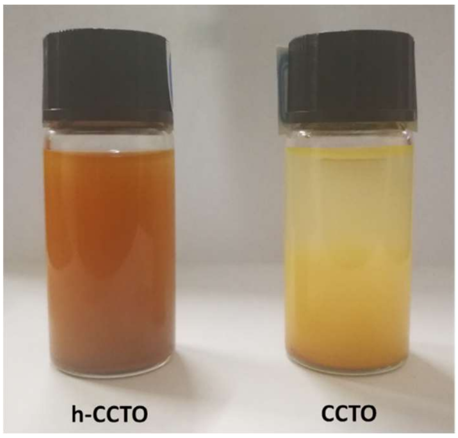

Figure S3. Digital photo of dispersions of pure CCTO and h-CCTO particles in ethanol after standing for four hours.

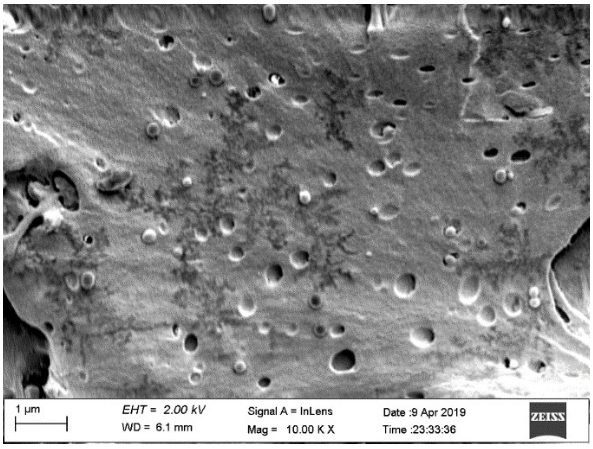

Figure S4. Cross-sectional SEM image of PAEN composites with 15 wt% non-hydroxylated CCTO particles.

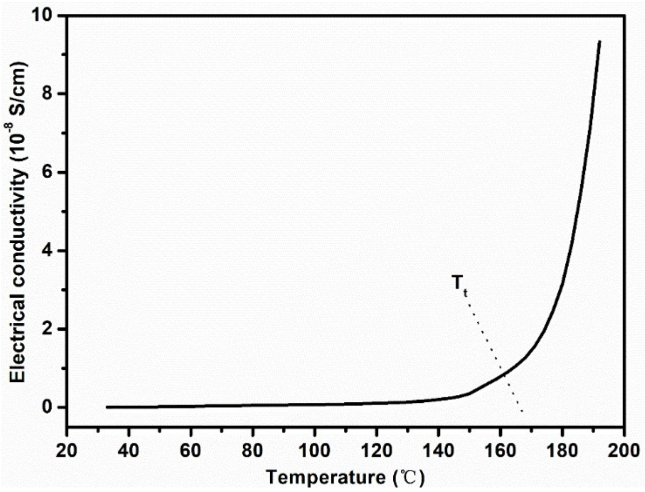

Figure S5. Electrical conductivity of PAEN/h-CCTO composite with 30 wt% h-CCTO content as a function of temperature.

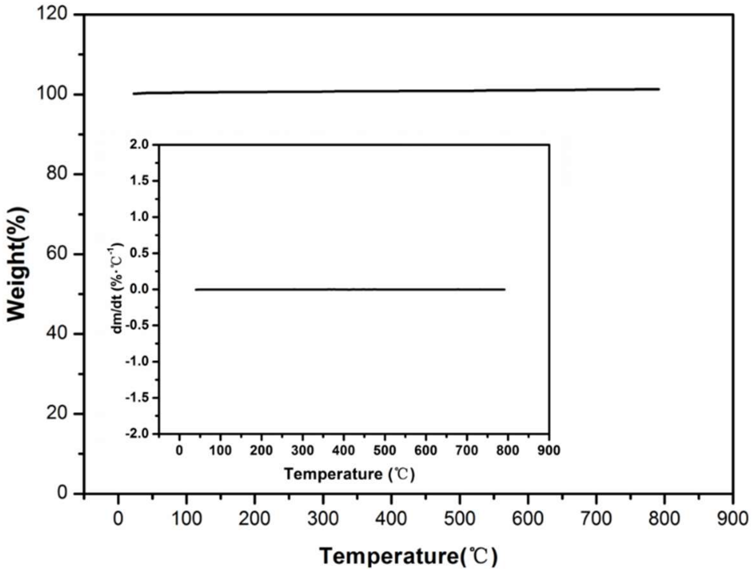

Figure S6. TGA and DTG curves of h-CCTO particles.

Reference:

1. Zheng, Y.; Wang, S.; Feng, J.; Ouyang, Z.; Li, X. Measurement of the complex permittivity of dry rocks and minerals: application of polythene dilution method and Lichtenecker's mixture formulae. *Geophys. J. Int.* **2005**, *163*, 1195–1202.
